# Serum magnesium, mortality and disease progression in chronic kidney disease

**DOI:** 10.1186/s12882-020-1713-3

**Published:** 2020-02-12

**Authors:** Rami Azem, Remy Daou, Elias Bassil, Eva Maria Anvari, Jonathan J. Taliercio, Susana Arrigain, Jesse D. Schold, Tushar Vachharajani, Joseph Nally, Georges N. Na khoul

**Affiliations:** 1 Department of Nephrology and Hypertension, Glickman Urological and Kidney Institute, Cleveland Clinic, 9500 Euclid Avenue - Q7, Cleveland, OH 44195 USA; 2 Department of Family Medicine, Saint Joseph University, Beirut, Lebanon; 3 Department of Internal Medicine, Cleveland Clinic, Cleveland, OH USA; 4 Cleveland Clinic Lerner College of Medicine of Case Western Reserve University, Cleveland, OH USA; 5 Department of Quantitative Health Sciences, Cleveland Clinic, Cleveland, OH USA

**Keywords:** Magnesium, CKD, Disease progression, Mortality

## Abstract

**Introduction:**

Magnesium disorders are commonly encountered in chronic kidney disease (CKD) and are typically a consequence of decreased kidney function or frequently prescribed medications such as diuretics and proton pump inhibitors. While hypomagnesemia has been linked with increased mortality, the association between elevated magnesium levels and mortality is not clearly defined. Additionally, associations between magnesium disorders, type of death, and CKD progression have not been reported. Therefore, we studied the associations between magnesium levels, CKD progression, mortality, and cause specific deaths in patients with CKD.

**Methods:**

Using the Cleveland Clinic CKD registry, we identified 10,568 patients with estimated Glomerular Filtration Rate (eGFR) between 15 and 59 ml/min/1.73 m^2^ in this range for a minimum of 3 months with a measured magnesium level. We categorized subjects into 3 groups based on these magnesium levels (≤ 1.7, 1.7–2.6 and > 2.6 mg/dl) and applied cox regression modeling and competing risk models to identify associations with overall and cause-specific mortality. We also evaluated the association between magnesium level and slope of eGFR using mixed models.

**Results:**

During a median follow-up of 3.7 years, 4656 (44%) patients died. After adjusting for relevant covariates, a magnesium level < 1.7 mg/dl (vs. 1.7–2.6 mg/dl) was associated with higher overall mortality (HR = 1.14, 95% CI: 1.04, 1.24), and with higher sub-distribution hazards for non-cardiovascular non-malignancy mortality (HR = 1.29, 95% CI: 1.12, 1.49). Magnesium levels > 2.6 mg/dl (vs. 1.7–2.6 mg/dl) was associated with a higher risk of all-cause death only (HR = 1.23, 95% CI: 1.03, 1.48). We found similar results when evaluating magnesium as a continuous measure. There were no significant differences in the slope of eGFR across all three magnesium groups (*p* = 0.10).

**Conclusions:**

In patients with CKD stage 3 and 4, hypomagnesemia was associated with higher all-cause and non-cardiovascular non-malignancy mortality. Hypermagnesemia was associated with higher all-cause mortality. Neither hypo nor hypermagnesemia were associated with an increased risk of CKD progression.

## Introduction

Magnesium is the second most abundant intracellular electrolyte and plays a significant role in essentially every biologic function within the cell [1]. It is important for bone and mineral metabolism, as well as for regulating vascular tone and heart rhythm [2, 3]. Magnesium does not appear to be controlled by any hormonal systems. Rather, the regulation of magnesium balance is dependent on intestinal absorption and renal excretion. Given the essential role of the kidneys in maintaining magnesium homeostasis, abnormalities in magnesium levels, including hypomagnesemia and hypermagnesemia, are not uncommon in patients with chronic kidney disease (CKD).

Hypomagnesemia in CKD is usually the result of medication use [4] such as diuretics, calcineurin inhibitors or proton pump inhibitors [5] but it can also be caused by associated conditions like diabetes or volume expansion [6]. Hypomagnesemia has been investigated in non-CKD, CKD, and end stage renal disease (ESRD) patient populations and has been found to be associated with increased mortality [1, 7, 8], notably increased cardiovascular mortality [9]. Hypomagnesemia has also been linked to a worsening rate of decline in eGFR, though this has not been clearly established [7, 10].

Hypermagnesemia in CKD is the result of decreased Glomerular Filtration Rate (GFR). Since urinary excretion is the primary magnesium regulatory system, plasma magnesium levels rise as renal function decreases [11]. The relationship between hypermagnesemia and mortality is not as clear. Some studies suggest that mild elevations in serum magnesium levels are associated with a survival advantage [7, 12–14]. There are no studies examining the associations between hypermagnesemia and CKD progression.

Our study examines the association between serum magnesium levels, all-cause mortality and progression of CKD in a large cohort of CKD patients.

## Materials and methods

### Patient population

We used the Cleveland Clinic CKD registry to evaluate the relationship between serum magnesium and outcomes. The CKD registry was developed from the electronic health records (EHR) and we have described the development and validation in detail in previously published work [15]. For this analysis, we included patients fulfilling the following criteria: a) had at least one in-person outpatient encounter with a Cleveland Clinic health care provider and at least two estimated glomerular fitration rate (eGFR) results < 60 ml/min/1.73 m^2^ taken more than 90 days apart from 2005 to 2014. (the second eGFR was 15–59.9 ml/min/1.73 m^2^ and patients were not on dialysis nor had a prior kidney transplant), b) had serum magnesium measured within the year prior to the date of second eGFR< 60 (CKD); and c) were residents of Ohio.

### Patient characteristics

We extracted demographic information including sex, age, race, and insurance from the EHR. We used previously specified and validated criteria to define comorbidities including hypertension, diabetes mellitus, coronary artery disease, congestive heart failure, hyperlipidemia, and malignancy [15]. Baseline conditions were ascertained prior to the second eGFR < 60 ml/min/1.73 m^2^. We also obtained laboratory measurements (serum hemoglobin, albumin, bicarbonate and potassium) from the EHR. For laboratory results other than serum magnesium, we used the last outpatient laboratory result within 2 years prior to second eGFR < 60 ml/min/1.73 m^2^.

### Kidney function

Our hospital measured serum creatinine in a clinical laboratory with a Hitachi D 2400 Modular Chemistry Analyzer (Roche Diagnostics, Indianapolis, IN). We used the CKD-EPI equation [16] to calculate eGFR. We classified CKD into the following stages: CKD stage 3a (eGFR 45–59 ml/min/1.73 m^2^), stage 3b (eGFR 30–44 ml/min/1.73 m^2^), and stage 4 CKD (eGFR 15–29 ml/min/1.73 m^2^)..

### Serum magnesium

Serum magnesium was measured using the Gen.2 homogeneous enzymatic calorimetric assay on the Cobas C702 analyzer (Roche diagnostics). Serum magnesium was classified based on the normal ranged provided by the assay into the following categories: < 1.7, 1.7–2.6, and > 2.6 mg/dl. Magnesium data obtained within 1 year prior to second eGFR < 60 ml/min/1.73 m^2^ was included.

### Ascertainment of death and its causes

We obtained mortality data from the EHR and through linkage of our registry with the Ohio Department of Health death records. The state death records contain causes of death coded according to the *International Classification of Diseases*, Tenth Revision (ICD-10). We grouped the underlying causes of death following the National Center for Health Statistics coding system, except for a modification explained here. We classified deaths into three categories: a) deaths from cardiovascular causes, b) deaths from malignancy, and c) deaths from other (non-cardiovascular and non-malignancy-related) conditions. We defined cardiovascular deaths as those due to diseases of the heart, essential hypertension, cerebrovascular disease, atherosclerosis, or other diseases of the circulatory system (ICD-10 codes I00–I78). Patients with death reported in the EHR but not found in Ohio mortality files were included in analyses of all-cause mortality and excluded from cause-specific analyses.

### Statistical analysis

We compared baseline characteristics between patients with and without magnesium data, and also among patients with different magnesium levels using Chi-square and ANOVA and Kruskal-Wallis tests for categorical and continuous variables, respectively. We summarized the leading causes of death for various magnesium categories as percent of total deaths observed. We estimated survival over time by magnesium level using Kaplan-Meier survival estimates. We evaluated the relationship between magnesium levels and overall mortality using a Cox proportional hazards model and the relationship between magnesium level and cause specific death categories using competing risks regression models as described by Fine and Gray. We adjusted the models for the following covariates: sex, age, race, diabetes, hyperlipidemia, hypertension, coronary artery disease, congestive heart failure, cerebrovascular disease, malignancy, body mass index, albumin, hemoglobin, calcium, serum bicarbonate, potassium, magnesium supplement, proton pump inhibitors, insurance group, ACEI (Angiotensin Conversion Enzyme Inhibitors) /ARB (Angiotensin Receptor Blockers), beta blockers, diuretics, smoking and eGFR. We used splines to relax linearity assumptions as necessary.

Approximately 5% of patients were missing Body Mass Index (BMI), albumin or hemoglobin data, < 0.1% were missing bicarbonate, potassium or calcium data, 23% were missing smoking status, and 6% did not have insurance data. We used multiple imputation (SAS proc. MI) to impute data in 2 steps. In the first step we used the Markov Chain Monte Carlo method and a single chain to impute 5 datasets with complete continuous and binary data. In the second step we imputed insurance group on each of the 5 datasets using discriminant function analysis^15^. We included the following covariates in the imputation: sex, age, race, diabetes, hyperlipidemia, hypertension, coronary artery disease, congestive heart failure, cerebrovascular disease, malignancy, body mass index, albumin, hemoglobin, calcium, serum bicarbonate, potassium, magnesium supplement, insurance group, ACEI/ARB, beta blockers, diuretics, smoking and eGFR. We ran all models on each of the 5 imputed datasets, and then combined parameter estimates using SAS MI analyze.

We also evaluated the association between continuous magnesium and all-cause mortality using restricted cubic splines at percentiles 10, 50 and 90 in the Cox model. We graphed continuous magnesium vs. the log hazard using data from the first imputation. The graph estimates were calculated for a hypothetical patient with mean values on all baseline covariates. We evaluated two-way interactions on all-cause mortality between magnesium level and each of the following: eGFR, BMI group and potassium level. We conducted a sensitivity analysis to confirm the main mortality findings while excluding patients with history of malignancy.

To evaluate the slope of eGFR at different values of baseline magnesium, we used a mixed model analysis with subjects considered a random effect and tested the interaction between magnesium level and time (slope of eGFR over time) until patients reached eGFR< 20 or dialysis initiation or preemptive transplant or end of study or end of follow up. We iteratively tested the covariance structure for mixed models and selected our final model based on best fit as determined by the Akaike Information Criteria. An autoregressive covariance structure was used for the final model. We used the first eGFR per month for each patient. We adjusted for the covariates mentioned above. Wed fit the model on each of the 5 imputed datasets, and parameter estimates were combined using SAS MI analyze.

All analyses were conducted using Linux SAS version 9.4 (SAS Institute, Cary, NC), and graphs were created using R 3.5.1 (The R Foundation for Statistical Computing, Vienna, Austria). This study and the CKD registry were both approved by the Cleveland Clinic Institutional Review Board (IRB study number 09–015).

## Results

### Patient characteristics

From 2005 to December 2014, there were 73,693 patients with CKD stage 3 or 4 in the registry who were residents of Ohio (Figure 3 in Appendix). Of those, 10,568 had magnesium levels within the year prior to entry into the registry and qualified for this study. Patients who had magnesium measured were younger, more male, more African American, had lower BMI, less diabetes, more malignancy, less hypertension, more congestive heart failure, and lower eGFR compared to those that didn’t have magnesium measured (Table 4 in Appendix). The mean age was 68.7 ± 13.3 years, 47% were men, and 14% were blacks. Mean BMI of the study cohort was 29.2 ± 7 kg/m^2^. Prevalence of diabetes, malignancy, and coronary artery disease were 21, 27 and 20% respectively. Mean eGFR of the study population was 46.3 ml/min/1.73 m^2^. Table 1 outlines other details of the study population based on magnesium categories.

**Table 1:** Patient characteristics by baseline Magnesium level

Factor	Overall (*N* = 10,568)	< 1.7 mg/dl (*N* = 1314)	1.7–2.6 mg/dl (*N* = 9049)	> 2.6 mg/dl (*N* = 205)	*p*-value
Age	68.7 ± 13.3	65.4 ± 13.5	69.1 ± 13.2	68.7 ± 14.0	< 0.001^a^
Male	4969 (47.0)	556 (42.3)	4309 (47.6)	104 (50.7)	< 0.001^c^
African American	1445 (13.7)	188 (14.3)	1222 (13.5)	35 (17.1)	0.26^c^
Smoking					0.005^c^
No	7414 (70.2)	884 (67.3)	6386 (70.6)	144 (70.2)	
Yes	723 (6.8)	122 (9.3)	588 (6.5)	13 (6.3)	
Missing	2431 (23.0)	308 (23.4)	2075 (22.9)	48 (23.4)	
BMI	29.2 ± 7.0	29.2 ± 7.1	29.2 ± 6.9	28.8 ± 8.0	0.80^a^
BMI group					0.20^c^
< 18.5 kg/m2	204 (1.9)	33 (2.5)	161 (1.8)	10 (4.9)	
18.5–24.9 kg/m2	2719 (25.7)	324 (24.7)	2343 (25.9)	52 (25.4)	
25–29.9 kg/m2	3291 (31.1)	409 (31.1)	2823 (31.2)	59 (28.8)	
30–34.9 kg/m2	2097 (19.8)	257 (19.6)	1801 (19.9)	39 (19.0)	
35–39.9 kg/m2	969 (9.2)	119 (9.1)	833 (9.2)	17 (8.3)	
40+ kg/m2	757 (7.2)	101 (7.7)	641 (7.1)	15 (7.3)	
Missing	531 (5.0)	71 (5.4)	447 (4.9)	13 (6.3)	
Diabetes	2175 (20.6)	316 (24.0)	1809 (20.0)	50 (24.4)	0.001^**c**^
Malignancy	2871 (27.2)	428 (32.6)	2405 (26.6)	38 (18.5)	< 0.001^**c**^
Hypertension	7998 (75.7)	1007 (76.6)	6835 (75.5)	156 (76.1)	0.68^**c**^
Hyperlipidemia	7303 (69.1)	849 (64.6)	6307 (69.7)	147 (71.7)	< 0.001^c^
CAD	2117 (20.0)	190 (14.5)	1875 (20.7)	52 (25.4)	< 0.001^c^
CHF	1589 (15.0)	107 (8.1)	1408 (15.6)	74 (36.1)	< 0.001^c^
CVD	894 (8.5)	83 (6.3)	796 (8.8)	15 (7.3)	0.009^c^
PVD	279 (2.6)	30 (2.3)	244 (2.7)	5 (2.4)	0.67^c^
ACEI/ARB	6025 (57.0)	688 (52.4)	5207 (57.5)	130 (63.4)	< 0.001^c^
Diuretics	6684 (63.2)	816 (62.1)	5715 (63.2)	153 (74.6)	0.002^c^
Statin	5138 (48.6)	578 (44.0)	4456 (49.2)	104 (50.7)	0.001^c^
Beta Blocker	5963 (56.4)	722 (54.9)	5113 (56.5)	128 (62.4)	0.12^c^
Magnesium supplement	2572 (24.3)	525 (40.0)	2012 (22.2)	35 (17.1)	< 0.001^c^
Proton pump inhibitor	5236 (49.5)	754 (57.4)	4390 (48.5)	92 (44.9)	< 0.001^c^
eGFR	46.3 ± 11.0	46.7 ± 10.4	46.3 ± 11.0	40.0 ± 12.9	< 0.001^a^
CKD stage					< 0.001^c^
45–59	6572 (62.2)	834 (63.5)	5652 (62.5)	86 (42.0)	
30–44	2888 (27.3)	367 (27.9)	2461 (27.2)	60 (29.3)	
15–29	1108 (10.5)	113 (8.6)	936 (10.3)	59 (28.8)	
Albumin	3.9 ± 0.59	3.8 ± 0.62	3.9 ± 0.59	3.9 ± 0.61	< 0.001^a^
Hemoglobin	12.3 ± 1.9	11.7 ± 1.9	12.4 ± 1.9	11.7 ± 2.2	< 0.001^a^
Potassium	4.3 ± 0.59	4.2 ± 0.64	4.3 ± 0.58	4.4 ± 0.64	< 0.001^a^
Calcium	9.4 ± 0.68	9.3 ± 0.76	9.4 ± 0.66	9.3 ± 0.86	< 0.001^a^
CO2	25.8 ± 3.8	25.0 ± 4.0	26.0 ± 3.7	26.3 ± 5.4	< 0.001^a^
Insurance grouped					0.035^c^
Medicaid	241 (2.3)	33 (2.5)	204 (2.3)	4 (2.0)	
Medicare	6901 (65.3)	821 (62.5)	5942 (65.7)	138 (67.3)	
Other	2835 (26.8)	367 (27.9)	2422 (26.8)	46 (22.4)	
Missing	591 (5.6)	93 (7.1)	481 (5.3)	17 (8.3)	

Statistics presented as Mean ± SD, or N (column %)

*p*-values: ^a^ANOVA, ^c^Pearson’s chi-square test

Number missing data: BMI 531, albumin 544, hemoglobin 529, potassium 6, calcium 5, CO2 6

### Mortality

During a median follow up of 3.7 years, 4656 (44%) patients died; cause of death was available for 4599 patients. Among them, 1597 (34.7%) died of cardiovascular causes, 1292 (28.1%) due to malignancy, 1623 (35.3%) due to non-cardiovascular non-malignancy diseases and 87 due to other causes. Table 2 shows the causes of death differed across magnesium levels (*p* < 0.001).

**Table 2:** Causes of death by baseline Magnesium level

	Total (*N* = 4599)	< 1.7 mg/dl (*N* = 628)	1.7–2.6 mg/dl (*N* = 3854)	> 2.6 mg/dl (*N* = 117)	Chi-square *p*-value
Cause of death group					< 0.001
Malignant Neoplasms	1292 (28.1)	223 (35.5)	1052 (27.3)	17 (14.5)	
All Cardiovascular Diseases	1597 (34.7)	148 (23.6)	1400 (36.3)	49 (41.9)	
All Other Diseases	1623 (35.3)	246 (39.2)	1328 (34.5)	49 (41.9)	
Non-Disease related deaths	87 (1.9)	11 (1.8)	74 (1.9)	2 (1.7)	

Statistics presented as *N* (column %)

### Magnesium and overall and cause-specific death

**Fig. 1:**
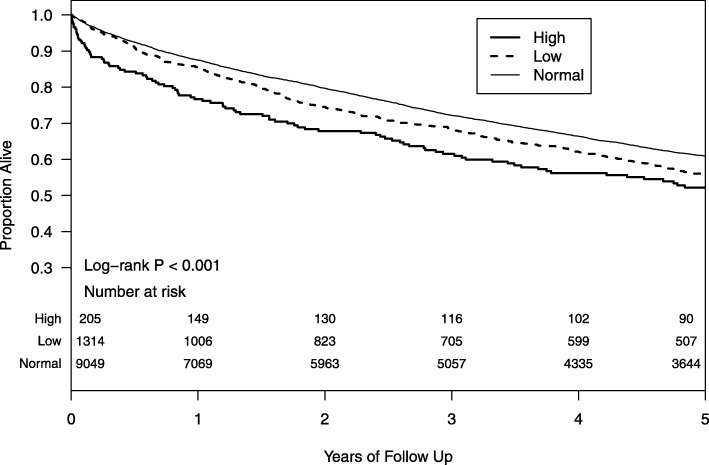
Kaplan-Meier survival estimates at 3 years for low, normal and high magnesium

**Fig. 2:**
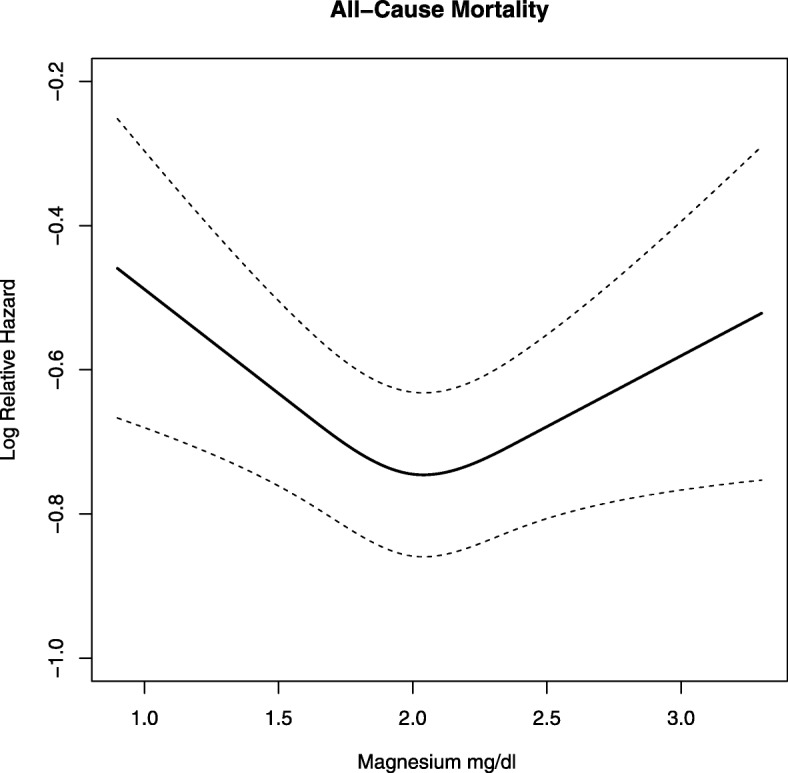
Relationship between magnesium (as a continuous measure) and all-cause mortality. The graph shows a U-shaped association between continuous magnesium and mortality

**Fig. 3:**
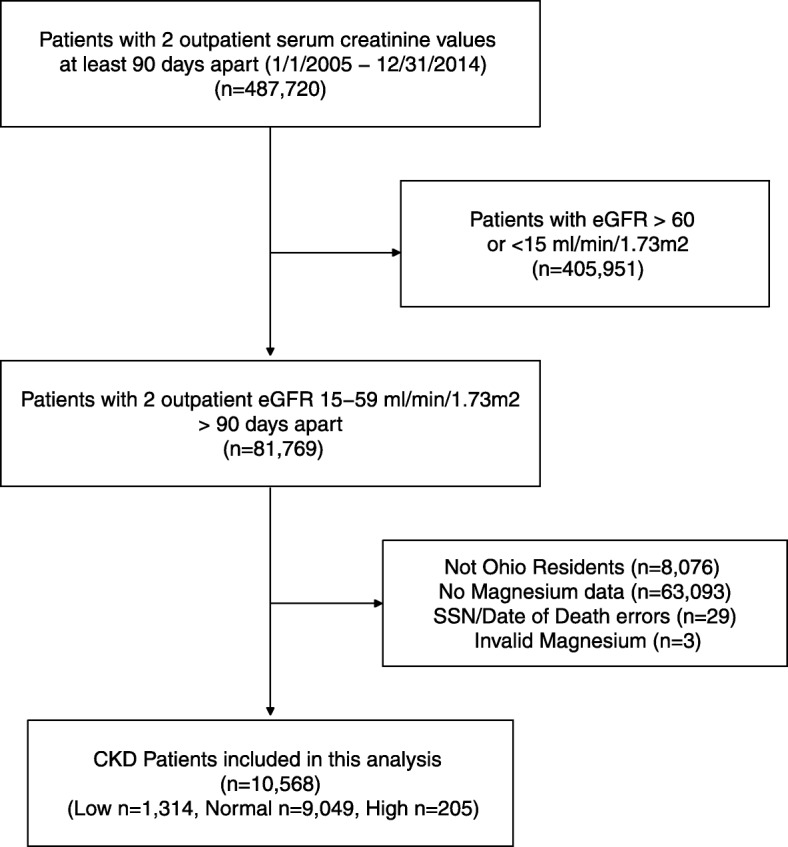
Flow chart showing how patients were selected for this analysis

Kaplan-Meier survival estimates at 3 years were 68.5% (95% CI: 65.9, 71.3), 72.2% (71.2, 73.2) and 61.5% (55.1, 68.7) for low, normal and high magnesium respectively (*P* < 0.001, Fig. 1). In the multivariable models adjusted for all relevant confounding variables, magnesium level < 1.7 mg/dl (vs. 1.7–2.6 mg/dl) was associated with higher overall mortality (HR = 1.14, 95% CI: 1.04, 1.24), and with higher sub-distribution hazards for non-cardiovascular non-malignancy mortality (HR = 1.29, 95% CI: 1.12, 1.49) (Table 3). Magnesium level > 2.6 mg/dl 1 (vs. 1.7–2.6 mg/dl) was associated with a higher risk of all-cause death only (HR = 1.23, 95% CI: 1.03, 1.48). In the model for all-cause death we found no evidence of an interaction between magnesium level and any of the following: eGFR, BMI group or potassium level. Figure 2 shows the associations between magnesium (as a continuous measure) and all-cause mortality.

**Table 3:** Association between magnesium level and mortality

Magnesium	All-cause unadjusted HR (95% CI)	All-cause HR (95% CI)*	Cardiovascular SHR (95% CI)*	Malignancy SHR (95% CI)*	Non-cardio non-malignancy causes SHR (95% CI)*
All patients					
< 1.7 mg/dl	1.14 (1.05, 1.24)	1.14 (1.04, 1.24)	0.91 (0.76, 1.08)	1.14 (0.97, 1.33)	1.29 (1.12, 1.49)
1.7–2.6 mg/dl	Ref	Ref	Ref	Ref	Ref
> 2.6 mg/dl	1.44 (1.20, 1.72)	1.23 (1.03, 1.48)	1.11 (0.81, 1.51)	0.71 (0.41, 1.22)	1.26 (0.93, 1.71)

*Adjusted for age, African American, male, diabetes, hyperlipidemia, BMI, albumin, hemoglobin, calcium, Acid/Base (CO2), potassium, magnesium supplement, malignancy, hypertension, CAD, CHF, cerebrovascular disease, Insurance group, Outpatient ACEI/ARB, Outpatient Beta Blocker, outpatient diuretics, proton pump inhibitors, smoking, eGFR

*All adjusted models used 5 datasets created with multiple imputation and MI analyze to obtain the HR or SHR

### Excluding patients with history of malignancy at baseline

In the sensitivity analyses excluding patients with malignancy (*n* = 7697), results were qualitatively similar to the primary analysis. Magnesium level < 1.7 mg/dl (vs. 1.7–2.6 mg/dl) was associated with higher overall mortality (HR = 1.16, 95% CI: 1.04, 1.29), and with higher sub-distribution hazards for non-cardiovascular non-malignancy mortality (HR = 1.37, 95% CI: 1.17, 1.61). Magnesium level > 2.6 mg/dl 1 (vs. 1.7–2.6 mg/dl) was associated with a higher risk of all-cause death only (HR = 1.27, 95% CI: 1.03, 1.55).

### eGFR decline

There were 7248 patients with follow up eGFR that were included in this analysis, 927 with low magnesium, 6188 normal and 133 high. No significant differences in the slope of eGFR were found among magnesium level groups (Interaction *P* = 0.10). The group with low magnesium had slightly more negative or deleterious monthly slope of eGFR than the normal group, but the effect was not statistically significant (Parameter estimate = − 0.02, SE = 0.01, *P* = 0.05).

## Discussion

In our study, we observed a U-shaped association between serum magnesium levels and mortality, with both hypomagnesemia and hypermagnesemia (HR = 1.23, 95% CI: 1.03, 1.48) demonstrating higher all-cause mortality. However, there was no association between serum magnesium levels and rate of eGFR decline.

Hypomagnesemia has been linked with increased all-cause mortality [7, 17]. In a recent meta-analysis of magnesium levels in CKD and ESRD patients, Xiong et al. reviewed 20 studies with more than 200,000 participants and showed a clear association between hypomagnesemia and all-cause mortality [18]. In particular, low magnesium seem to be associated with increased cardiovascular mortality and the proposed mechanisms include promoting hypertension, cardiac arrhythmias [19], cardiac remodeling / fibrosis [20], oxidative stress, insulin resistance and arterial stiffening [17, 21]. It has also been suggested that magnesium has anti-atherosclerotic effects, and that hypomagnesemia promotes calcification in vascular smooth muscle cells [22], as well as endothelial cell dysfunction [17]. Our findings are consistent with the literature whereas hypomagnesemia was indeed associated with increased mortality. However, we did not observe a higher risk of cardiovascular mortality specifically. This could be due to the fact that in our population, patients with hypomagnesemia were younger and had lower incidence of cardiovascular disease. Rather, we report a higher prevalence of non-cardiac, non-malignancy related death.

Conversely, the relationship between hypermagnesemia and mortality is not clearly established, whereas some studies suggest that hypermagnesemia confers a survival advantage in CKD patients, while others report an increase in mortality. An observational retrospective study of chronic hemodialysis patients suggested a survival advantage with slightly elevated serum magnesium levels, however, found that patients with significantly elevated magnesium levels ≥3.1 had higher mortality [17]. It is hypothesized that this may be due to over suppression of Parathyroid Hormone (PTH) with significant hypermagnesemia, and that iPTH < 50 pg/ml is associated with higher cardiovascular mortality [17]. This study did not include non-dialysis CKD patients, however. Other observational studies found that ESRD patients with arterial calcification, mitral annular calcification, and carotid intima-media thickness had lower serum magnesium levels on average, and therefore suggest that hypermagnesemia may play a protective role in this setting, however these studies did not examine survival [13]. However, other studies contradict those findings and show an association between hypermagnesemia and morbidity and mortality, notably in critically ill patients [23, 24]. Indeed, hypermagnesemia can lead to hypotension, cardiac conduction abnormalities, neuromuscular blockade and respiratory depression [25–27]. Our findings are in line with the latter set of studies whereas hypermagnesemia was associated with a higher prevalence of all-cause mortality. But there were no statistically significant differences in specific causes of death for patients with hypermagnesemia.

There are few studies that examine the effects of hypomagnesemia and hypermagnesemia on CKD progression. One study found that hypomagnesemia was associated with an annual eGFR decline of 9.6% when baseline serum magnesium level was 1 mg/dL below the population mean, 3.5% annual eGFR decline if the serum magnesium level was 1 mg/dL above the population mean, and 6.6% eGFR decline for the population mean, however the effect of magnesium lost significance after adjustment for covariates [17]. Proposed mechanisms for why hypomagnesemia may be responsible for accelerated eGFR decline include potentiating hyperaldosteronism, hypertension, endothelial dysfunction, and oxidative stress, which may lead to further kidney injury [17]. In our study, we compared the slope or rate of decline in eGFR between CKD patients with hypomagnesemia, normal magnesium levels, and hypermagnesemia and found that the association between magnesium group and slope of eGFR was not statistically significant in either the unadjusted (*P* = 0.14) or adjusted (*P* = 0.10) models, suggesting that different magnesium levels are not associated with a different rate of eGFR decline in CKD patients. However, we do note that the group with low magnesium levels had a slightly more negative slope than the normal group, but the effect was not statistically significant.

Strengths of our analysis include a large patient population with CKD stages 3–4, with data over several years, including a diverse patient population. However, retrospective analyses are prone to residual confounding. While we were able to control for multiple confounding variables that affect mortality, we were unable to adjust for patients on a calcimimetic, as well as for iPTH and phosphorous levels due to insufficient data. Additionally, we recognize that the patients who had their magnesium levels measured were different from those who did not and we realize this could bias our results. For example, serum magnesium appeared to be measured frequently in patients with malignancy and that could be driven by the known hypomagnesemic effects of several anti-cancer drugs. Unfortunately, we did not have data related to the use of cancer drugs, so we could not investigate this finding further. Finally, our patients have been followed in a single health care system, and therefore the data may not be generalizable.

## Conclusion

In conclusion, hypomagnesemia and hypermagnesemia were both associated with increased mortality, demonstrating a U-shaped association between serum magnesium levels and mortality. Hypomagnesemia was associated specifically associated with increased non-cardiovascular, non-malignancy related death while hypermagnesemia did not demonstrate any cause specific mortality association. Magnesium levels are not associated with a difference in rate of eGFR decline in CKD patients.

## Data Availability

Data supporting our findings is contained within the manuscript and the appendix. The totality of the data cannot be shared based on patient confidentiality concerns by which the IRB approved our CKD registry.

## Appendix

**Table 4:** Patient characteristics for those having magnesium measured vs. not measured

Factor	*N* missing	Overall (*N* = 73,542)	No Magnesium (*N* = 62,974)	Have Magnesium (*N* = 10,568)	*p*-value
Age	0	72.0 ± 11.8	72.6 ± 11.4	68.7 ± 13.3	< 0.001^a^
Male	0	32,180 (43.8)	27,211 (43.2)	4969 (47.0)	< 0.001^c^
African American	0	9255 (12.6)	7810 (12.4)	1445 (13.7)	< 0.001^c^
Smoking	0				< 0.001^c^
No		58,506 (79.6)	51,092 (81.1)	7414 (70.2)	
Yes		5182 (7.0)	4459 (7.1)	723 (6.8)	
Missing		9854 (13.4)	7423 (11.8)	2431 (23.0)	
BMI	2532	29.6 ± 6.6	29.7 ± 6.6	29.2 ± 7.0	< 0.001^a^
BMI group	0				< 0.001^c^
< 18.5 kg/m2		963 (1.3)	759 (1.2)	204 (1.9)	
18.5–24.9 kg/m2		16,508 (22.4)	13,789 (21.9)	2719 (25.7)	
25–29.9 kg/m2		25,009 (34.0)	21,718 (34.5)	3291 (31.1)	
30–34.9 kg/m2		15,962 (21.7)	13,865 (22.0)	2097 (19.8)	
35–39.9 kg/m2		7281 (9.9)	6312 (10.0)	969 (9.2)	
40+ kg/m2		5287 (7.2)	4530 (7.2)	757 (7.2)	
Missing		2532 (3.4)	2001 (3.2)	531 (5.0)	
Diabetes	0	17,409 (23.7)	15,234 (24.2)	2175 (20.6)	< 0.001^c^
Malignancy	0	18,226 (24.8)	15,355 (24.4)	2871 (27.2)	< 0.001^c^
Hypertension	0	62,427 (84.9)	54,429 (86.4)	7998 (75.7)	< 0.001^c^
Hyperlipidemia	0	56,763 (77.2)	49,460 (78.5)	7303 (69.1)	< 0.001^c^
CAD	0	14,958 (20.3)	12,841 (20.4)	2117 (20.0)	0.40^c^
CHF	0	5964 (8.1)	4375 (6.9)	1589 (15.0)	< 0.001^c^
CVD	0	6810 (9.3)	5916 (9.4)	894 (8.5)	0.002^c^
PVD	0	2432 (3.3)	2153 (3.4)	279 (2.6)	< 0.001^c^
ACEI/ARB	0	46,076 (62.7)	40,051 (63.6)	6025 (57.0)	< 0.001^c^
Diuretics	0	47,685 (64.8)	41,001 (65.1)	6684 (63.2)	< 0.001^c^
Statin	0	42,014 (57.1)	36,876 (58.6)	5138 (48.6)	< 0.001^c^
Beta Blocker	0	40,438 (55.0)	34,475 (54.7)	5963 (56.4)	0.001^c^
Magnesium supplement	0	7156 (9.7)	4584 (7.3)	2572 (24.3)	< 0.001^c^
Proton pump inhibitor	0	31,152 (42.4)	25,916 (41.2)	5236 (49.5)	< 0.001^c^
eGFR	0	47.8 ± 10.2	48.1 ± 10.1	46.3 ± 11.0	< 0.001^a^
CKD stage	0				< 0.001^c^
45–59		50,169 (68.2)	43,597 (69.2)	6572 (62.2)	
30–44		17,765 (24.2)	14,877 (23.6)	2888 (27.3)	
15–29		5608 (7.6)	4500 (7.1)	1108 (10.5)	
Albumin	10,714	4.1 ± 0.46	4.1 ± 0.42	3.9 ± 0.59	< 0.001^a^
Hemoglobin	12,475	12.8 ± 1.8	12.9 ± 1.8	12.3 ± 1.9	< 0.001^a^
Potassium	684	4.3 ± 0.53	4.3 ± 0.52	4.3 ± 0.59	< 0.001^a^
Calcium	770	9.5 ± 0.58	9.6 ± 0.56	9.4 ± 0.68	< 0.001^a^
CO2	751	25.9 ± 3.3	25.9 ± 3.2	25.8 ± 3.8	0.003^a^
Insurance grouped	0				< 0.001^c^
Medicaid		1320 (1.8)	1079 (1.7)	241 (2.3)	
Medicare		50,892 (69.2)	43,991 (69.9)	6901 (65.3)	
Missing		2796 (3.8)	2205 (3.5)	591 (5.6)	
Other		18,534 (25.2)	15,699 (24.9)	2835 (26.8)	

Statistics presented as Mean ± SD, or N (column %)

*p*-values: ^a^ANOVA, ^c^Pearson’s chi-square test

## Abbreviations

ACEI: Angiotensin Conversion Enzyme Inhibitors
ARB: Angiotensin Receptor Blockers
BMI: Body Mass Index
CAD: Coronary Artery Disease
CHF: Congestive Heart Failure
CKD: Chronic Kidney disease
CVD: Cerebro-Vascular Disease
eGFR: Estimated Glomerular Filtration Rate
EHR: Electronic Health Records
ICD-10: International Classification of Diseases*,* Tenth Revision
IRB: Institutional Review Board
PTH: Parathyroid Hormone
PVD: Peripheral Vascular Disease
