# Correction to: Serum magnesium, mortality and disease progression in chronic kidney disease

**DOI:** 10.1186/s12882-020-01801-0

**Published:** 2020-04-17

**Authors:** Rami Azem, Remy Daou, Elias Bassil, Eva Maria Anvari, Jonathan J. Taliercio, Susana Arrigain, Jesse D. Schold, Tushar Vachharajani, Joseph Nally, Georges N. Nakhoul

**Affiliations:** 1 Department of Nephrology and Hypertension, Glickman Urological and Kidney Institute, Cleveland Clinic, 9500 Euclid Avenue - Q7, Cleveland, OH 44195 USA; 2 Department of Family Medicine, Saint Joseph University, Beirut, Lebanon; 3 Department of Internal Medicine, Cleveland Clinic, Cleveland, OH USA; 4 Cleveland Clinic Lerner College of Medicine of Case Western Reserve University, Cleveland, OH USA; 5 Department of Quantitative Health Sciences, Cleveland Clinic, Cleveland, OH USA

**Correction to: BMC Nephrology (2020) 21:49.**

**https://doi.org/10.1186/s12882-020-1713-3**

Following publication of the original article [1], we have been notified that the name of one author was spelled incorrectly as Georges N. Na khoul, when the correct spelling is Georges N. Nakhoul.
